# Platelet RNA-Seq Reveals Genes Associated with Carotid Intima-Media Thickness: A Cross-Sectional Study

**DOI:** 10.1055/a-2661-6472

**Published:** 2025-08-07

**Authors:** Zhanfei Tan, Fan Guo, Jiaming Gao, Lanlan Li, Shujuan Xu, Yehao Zhang, Jianhua Fu, Jianxun Liu

**Affiliations:** 1 Wangjing Hospital, China Academy of Chinese Medical Sciences, Beijing, China; 2 Institute of Basic Medical Sciences of Xiyuan Hospital, China Academy of Chinese Medical Sciences, Beijing, China; 3 Beijing Key Laboratory of Chinese Materia Pharmacology, Beijing, China

**Keywords:** atherosclerosis, *PF4*, platelet, RNA sequencing, WGCNA

## Abstract

**Background:**

Although the association between platelet characteristics and the risk of developing atherosclerosis (AS) has been acknowledged, the specific role of platelets in AS development and progression remains unclear. Therefore, the aim of this study was to identify platelet characteristics in patients with and without AS to enhance the understanding of their pathophysiological functions and discover more sensitive biomarkers for AS diagnosis.

**Methods:**

We conducted a cross-sectional study involving AS patients and healthy controls (N). Based on the Chinese guidelines for diagnosing carotid and vertebral artery AS and the 2010 American College of Cardiology Foundation/American Heart Association (ACCF/AHA) guidelines, we defined AS using carotid ultrasound to measure intima-media thickness (IMT). General information, including sex, age, height, and weight, was collected upon enrollment. A series of examinations, including physical exams, serum lipid profiles, blood glucose tests, liver and kidney function tests, platelet aggregation assays, and carotid artery ultrasounds, was performed. Platelets were extracted from plasma for RNA-seq analysis.

**Results:**

No statistically significant differences in age, sex, body mass index, or blood pressure were observed between the groups. Total triglyceride, total cholesterol, low-density lipoprotein cholesterol, apolipoprotein B, red blood cell count, hemoglobin concentration, cholesterol levels, and carotid IMT were significantly greater, and vascular endothelial function was significantly lower in the AS group than in the N group. Using RNA-seq, we identified 784 differentially expressed genes—141 downregulated and 643 upregulated—with Gene Ontology enrichment showing significant associations with blood coagulation pathways, among others. Weighted correlation network analysis revealed four hub genes related to IMT: *Integrin Subunit Alpha 2b (ITGA2B), Transforming Growth Factor Beta 1 (TGFB1), Platelet Factor 4 (PF4)* , and *Glycoprotein IX Platelet (GP9)* .

**Conclusion:**

Our findings indicate moderate correlations of elevated *ITGA2B* ( *r* = 0.327, *p* = 0.004), *TGFB1* ( *r* = 0.362, *p* = 0.001), *PF4* ( *r* = 0.240, *p* = 0.038), and *GP9* ( *r* = 0.302, *p* = 0.008) levels with increased IMT, suggesting that these genes may serve as predictive biomarkers for AS.

## Introduction

Atherosclerosis (AS) is a lipid-driven chronic progressive inflammatory disease that affects mainly large- and medium-sized arteries. 1 It is characterized by the formation of fibrous or atheromatous plaques in the intima of blood vessels, leading to stenosis of the lumen and weakening of the elasticity of the wall, which affects blood perfusion in the corresponding tissues and organs. 1 2 In addition, plaque rupture and subsequent occlusive thrombosis may be the major direct triggers of most acute myocardial ischemic events, such as unstable angina and acute myocardial infarction. 3 4

The currently available literature has revealed that platelets play an important role in the pathogenesis and progression of AS. 5 First, as a major risk factor for AS, hyperlipidemia has long been considered primarily responsible for the development of AS. 6 7 Research has shown that hyperlipidemia significantly increases the biogenesis, turnover, and activity of platelets. 8 Second, AS is a chronic inflammatory disease, and the slow formation process of AS lesions involves the participation of many bone marrow and immune cells (including neutrophils, eosinophils, monocytes, and lymphocytes) 1 ; moreover, platelets seem to play a key role in the recruitment of these inflammatory effector cells, and the crosstalk between platelets and these inflammatory cells regulates the progression of inflammation. 9 10 11 12 Finally, the clinical application of antiplatelet therapy can effectively prevent the formation of new lesions and stabilize existing lesions in patients with AS. 13 14

The current diagnostic workflow for AS heavily relies on traditional markers such as lipid profiles and carotid artery ultrasound imaging. However, these methods, while important, do not fully capture the complexity of AS pathophysiology. This limitation has hindered the identification of highly sensitive biomarkers that could enable early detection and more accurate risk stratification. Therefore, the aim of this study was to explore novel platelet biomarkers that could enhance the diagnostic accuracy for AS, potentially leading to better clinical management of the disease. While previous studies have demonstrated that platelet characteristics play a role in the development of AS, the mechanisms remain unclear. Research has shown that platelet activation and aggregation are crucial in AS progression, yet these processes are not sufficiently integrated into the current diagnostic approach. 15 16 By focusing on RNA-seq of platelets from AS patients, we aim to uncover gene expression signatures that could serve as early diagnostic markers, improving upon current diagnostic practices. In the present study, we found that the expression levels of the *ITGA2B* , *TGFB1* , *PF4* , and *GP9* genes were significantly correlated with the intima-media thickness (IMT), suggesting potential avenues for the future prediction or diagnosis of AS.

While previous studies have demonstrated that platelet characteristics play a role in the development of AS, the mechanisms remain unclear. Research has shown that platelet activation and aggregation are crucial in AS progression, yet these processes are not sufficiently integrated into the current diagnostic approach (Zhu et al., 2018 3 ; Wang and Tang, 2020 7 ). By focusing on RNA-seq of platelets from AS patients, we aim to uncover gene expression signatures that could serve as early diagnostic markers, improving upon current diagnostic practices.

The aim of this study was to investigate the differential gene expression in platelets between these two groups. To achieve this, we recruited healthy subjects and AS patients and applied parametric transcriptome sequencing. Our findings may provide valuable insights for clinicians and researchers in elucidating the role of platelets in AS pathogenesis at the global transcriptional level, as well as identifying potential targets for effective interventions.

## Methods

### General Information

Eighty-five consecutive patients with AS admitted to the outpatient clinic and wards of Xi Yuan Hospital, China Academy of Chinese Medical Sciences, from September 2019 to September 2020, were retrospectively divided into a normal group (N) and an AS group. The study was approved by the Ethics Committee of Xi Yuan Hospital, China Academy of Chinese Medical Sciences (2020XLA057).

### Diagnostic Criteria

According to the 2010 American College of Cardiology Foundation/American Heart Association (ACCF/AHA) guidelines and the Chinese Guidelines for the Diagnosis of Carotid Atherosclerosis, 17 the diagnosis of AS was based on the measurement of IMT using carotid ultrasound. An IMT of less than 1.0 mm was considered normal, an IMT between 1.0 and 1.4 mm was classified as thickening, and an IMT greater than 1.5 mm or the presence of plaques was diagnostic of carotid plaque formation.

### Inclusion Criteria

The inclusion criterion for AS patients was as follows: Age between 20 and 65 years (including both boundary ages and applicable to both sexes); IMT >1.0 mm or visible plaque who without prior use of lipid-lowering drugs, antiplatelet drugs, and other medications that could potentially affect the experimental results. The inclusion criteria for Group N were the same as those for the AS group (age between 20 and 65 years [including both boundary ages and applicable to both sexes]), with the addition of general physical examination, liver and kidney function tests, blood lipid analysis, blood tests, urine tests, and routine stool tests—all yielding normal results. No participant had been involved in any other drug clinical trials within the past month. All the subjects participated voluntarily and signed informed consent forms.

### Exclusion Criteria

Those who had received lipid-lowering or anti-AS medications within the past 3 months, were currently receiving drug treatment for other medical conditions, or had medication-induced dyslipidemia, as well as those who had known conditions that could affect IMT measurements, such as hypertension, coronary artery disease, chronic kidney disease, or diabetes, were excluded from the study because of their inability to fully cooperate in completing all examinations and scales.

### Physical Examination and Laboratory Tests

In the present study, we collected comprehensive demographic information, disease history, medication history, diet and daily living conditions, gastrointestinal status, daily exercise, and other relevant information from the subjects. Blood pressure was measured by the doctor via a standard mercury sphygmograph after the patient had been seated for 5 minutes. Blood samples were collected in the fasting state at 8 a.m. After allowing the peripheral blood samples to stand at room temperature for half an hour, the serum samples were centrifuged at 3,000 rpm for 5 minutes before being extracted, packaged, and stored at −80 °C. Samples were promptly sent to our hospital's clinical laboratory department to assess the following clinical laboratory parameters: Routine blood analysis, lipid profile assessment, liver and kidney function evaluation, glucose measurements, and platelet aggregation tests. Additional types of clinical information, such as defecation status, dietary habits, daily living conditions, gastrointestinal status, disease history, and medication history, were obtained through face-to-face questionnaire surveys conducted by visiting physicians.

### Platelet Aggregation Test

Blood was collected from healthy and AS individuals via venipuncture and stored in vacuum-anticoagulated tubes containing lithium heparin. The whole blood was immobilized at 37 °C for 10 minutes before platelet-rich plasma extraction was performed. Anticoagulated whole blood was centrifuged at 300 × g for 7 minutes to obtain platelet-rich plasma. Platelet aggregation was induced by adding activators at 37 °C with a platelet aggregator (Chrono-Log Corp, Havertown) with sample agitation set at 1,000 rpm. Human platelet-rich plasma was activated with ADP (2.5 μM) and collagen (5 μg/mL). Gel-filtered platelets were activated with collagen (10 μg/mL) and thrombin (0.5 U). Additionally, the effects of human recombinant apolipoprotein A-IV on platelet aggregation induced by different agonists were measured after incubation for 2 minutes. Changes in transmittance resulting from platelet aggregation were detected and recorded for a minimum of 10 minutes.

### Carotid Ultrasound Examination

The proximal end of the common carotid artery was initially examined, followed by scanning of the internal and external carotid arteries along the vessel. The arterial IMT was measured, and any local protrusions were observed. The maximum size of each plaque was documented. Carotid ultrasonography was performed with a color Doppler ultrasound diagnostic instrument (Mindray M7, China) with a probe frequency of 10 MHz. The procedure involved two individuals; one conducted the examination, while the other recorded and entered the inspection results.

### RNA-Seq of Platelets

Total RNA was extracted from control and AS platelet samples, and RNA sequencing was performed via OEBiotech (Shanghai, China). Firstly, multivariate statistical analysis was conducted on the obtained gene expression data to elucidate intragroup repeatability and intergroup differences, as well as to ensure the reliability of sequencing results. The orthogonal partial least squares-discriminant analysis (OPLS-DA) method was employed in this study. Differentially expressed genes (DEGs) were screened and identified mainly through log2 (fold change) and *p* -values. Finally, the DEGs were characterized via Gene Set Enrichment Analysis (GSEA), Kyoto Encyclopedia of Genes and Genomes (KEGG) pathway analysis, and protein–protein interaction (PPI) network analysis.

### Weighted Correlation Network Analysis and Pearson's Correlation Analysis

The gene coexpression network was constructed using OECloud tools, and hierarchical clustering analysis was performed based on weighted correlation. The resulting clusters were segmented according to predefined criteria to obtain distinct gene modules, which were visually represented by branches and different colors in the cluster tree. Subsequently, the modules with higher correlation coefficients were identified by calculating the correlation between gene modules and IMT.

### Statistics

SPSS 25.0 software (IBM) was used for data analysis and processing. Count data were expressed as the number of cases and percentages, and the χ ^2^ test was used for comparisons between groups. Measurement data that conformed to a normal distribution were expressed as the mean ± standard deviation (x), and independent-samples *t* -tests were used for comparisons between two groups. A non-parametric test was used for non-normally distributed data. General linear bivariate Pearson's linear correlation analysis was used to examine the associations. Differences were considered statistically significant when *p* < 0.05.

## Results

### Baseline Characteristics of the Study Participants

A total of 41 normal individuals (N) and 40 AS patients were included in the study. There were no significant differences in age, sex, body mass index, systolic blood pressure, or diastolic blood pressure between the two groups ( *p* > 0.05; Table 1 ).

**Table 1:** Baseline characteristics of study participants

Variable	N ( *n* = 41)	AS ( *n* = 40)	*p* -Value
Age, years	48.27 ± 7.88	51.48 ± 8.49	0.082
Male, number (%)	10 (24.4%)	18(45.0%)	0.051
Body mass index, kg/m ^2^	23.94 ± 2.25	23.90 ± 2.17	0.924
Systolic pressure (mm Hg)	120.00 (10.00)	126.50 (19.50)	0.143
Diastolic pressure (mm Hg)	80.00 (0)	80.00 (5.00)	0.417

Abbreviations: AS, atherosclerosis group; N, control group.

### Carotid Ultrasonography Revealed Significant Differences in IMT and Flow-Mediated Dilation between the Two Groups

Among the 40 patients included in the AS group, visible plaques were detected in 21 patients. In this study, none of the 40 AS patients included had a carotid ultrasound report showing an IMT >1.5 mm, although the ultrasound results did report the presence and size of plaques (detailed data can be found in Supplementary Table S1 ). The echocardiography results revealed arterial stiffening and decreased vascular elasticity and compliance in the AS group. Specifically, carotid IMT was significantly greater ( *p* < 0.01) and vascular endothelial function (flow-mediated dilation; FMD) was significantly lower ( *p* < 0.05) in the experimental group than in the N group. There were no significant differences observed between the two groups regarding elasticity parameters, arterial compliance, arterial index, stiffness index beta, or aortic index ( *p* > 0.05; Table 2 ).

**Table 2:** Ultrasound assessment of carotid arteries

Variable	N ( *n* = 41)	AS ( *n* = 40)	*p* -Value
Left			
IMT (mm)	0.56 (0.14)	1.04 (0.55)	0.005 a
Ep (N/m ^2^ )	95.0 (41.50)	99.00 (105.00)	0.501
AC (mL/mm Hg)	0.72 (0.41)	0.71 (0.32)	0.951
AI	12.10 (14.90)	13.7 (13.60)	0.781
PMVβ (m/s)	5.90 (1.15)	6.10 (3.50)	0.501
β (cm/s)	6.90 (2.60)	6.70 (5.20)	0.801
Right			
IMT (mm)	0.56 (0.14)	1.04 (0.49)	<0.001 b
Ep (N/m ^2^ )	91.00 (47.00)	98.00 (40.75)	0.143
AC (mL/mm Hg)	0.81 ± 0.29	0.77 ± 0.26	0.591
AI	12.11 ± 9.04	10.20 ± 11.08	0.476
PMVβ (m/s)	12.20 (11.25)	11.75 (12.28)	0.971
β (cm/s)	5.60 (1.35)	6.05 (1.33)	0.062
FMD (%)	10.9 (6.81)	5.48 (6.33)	0.024 c

Abbreviations: AC, arterial compliance; AI, arterial index; AS, atherosclerosis group; Ep, elasticity parameters; FMD, flow-mediated dilation; IMT, intima-media thickness; N, control group; PMVβ, aortic index; β, stiffness index beta.

a *p* < 0.1.

b *p* < 0.001.

c *p* < 0.5.

### Comparison of Laboratory Test Results between the Two Groups

Blood lipids, blood glucose, liver and kidney function, coagulation function, and platelet aggregation test results were compared between the two groups. The results of the experiment showed that the levels of triglyceride, total cholesterol, low-density lipoprotein cholesterol (LDL-C), apolipoprotein B, red blood cells (RBCs), and hemoglobin concentration (HGB) in the AS group were significantly greater than those in the N group ( *p* < 0.05), whereas no significant differences were detected in the other test results between the two groups ( *p* > 0.05; Table 3 ).

**Table 3:** Comparison of blood fat, blood glucose, renal and liver function tests, coagulation function test, and platelet aggregation test

Variable	N ( *n* = 41)	AS ( *n* = 40)	*p* -Value
GLU (mmol/L)	5.36 (0.67)	5.52 (0.71)	0.385
WBC (×10 ^9^ /L)	5.47 ± 1.41	5.95 ± 1.69	0.160
RBC (×10 ^12^ /L)	4.25 (0.44)	4.54 (0.50)	0.012 a
HGB (g/L)	137.00 (13.75)	146.50 (17.50)	0.045 b
LY (%)	33.58 ± 6.52	33.51 ± 8.53	0.964
NEUT (%)	58.96 ± 6.86	58.51 ± 8.50	0.794
PLT (×10 ^9^ /L)	248.34 ± 45.46	244.90 ± 54.00	0.757
MONO (%)	5.41 ± 1.00	5.13 ± 0.93	0.196
TG (mmol/L)	0.91 (0.56)	1.15 (1.18)	0.024 b
TC (mmol/L)	4.67 (1.23)	5.11 (1.08)	0.007 a
HLDL-C (mmol/L)	1.30 (0.31)	1.19 (0.38)	0.388
LDL-C (mmol/L)	2.81 (1.01)	3.41 (1.16)	<0.001 c
VLDL (mmol/L)	0.52 (0.21)	0.45 (0.31)	0.362
ApoA (g/L)	1.12 ± 0.13	1.10 ± 0.12	0.357
ApoB (g/L)	0.80 (0.19)	0.92 (0.19)	<0.001 c
LP (A) (mg/dL)	87.21 (123.75)	115.57 (179.32)	0.134
ALT (U/L)	13.45 (5.80)	16.05 (11.50)	0.250
AST (U/L)	17.55 (6.70)	18.70 (8.88)	0.250
γ-GT (U/L)	15.09 (9.33)	18.45 (21.14)	0.050
UREA (mmol/L)	4.64 ± 1.05	5.03 ± 1.19	0.129
BUN (mg/dL)	12.75 ± 3.28	14.09 ± 3.33	0.076
CREA (μmol/L)	61.50 (9.75)	74.50 (16.50)	<0.001 c
UA (μmol/L)	272.46 ± 62.99	300.13 ± 79.15	0.092
PT (seconds)	11.10(0.475)	11.10(0.525)	0.877
PTA (%)	107.00 (6.00)	107.00(6.65)	0.657
INR	0.96 (0.04)	0.96 (0.04)	0.709
APTT (seconds)	27.85 (2.90)	27.90 (2.08)	0.795
TT (seconds)	17.20 (0.65)	17.20 (0.90)	0.661
Fbg (g/L)	2.69 (0.49)	2.81 (0.62)	0.378
PAg-AA (%)	80.10 (25.28)	78.88 (16.62)	0.988
PAg-ADP (%)	61.16 (38.78)	64.42 (46.05)	0.937
PAg-COL (%)	75.97 (31.54)	75.07 (31.33)	0.988

Abbreviations: ALT, alanine aminotransferase; ApoA, apolipoprotein A; ApoB, apolipoprotein B; APTT, activated partial thromboplastin time; AS, atherosclerosis group; AST, aspartate aminotransferase; BUN, blood urea nitrogen; CREA, creatinine; FBG, fibrinogen; GLU, blood glucose; HGB, hemoglobin; HLDL-C, high-density lipoprotein cholesterol; INR, prothrombin time ratio; LDL-C, low-density lipoprotein; LP (A), lipoprotein A; LY, lymphocyte; MONO, monocyte; N, control group; NEUT, neutrophil; PAg-AA, platelet maximal aggregation rate–arachidonic acid; PAg-ADP, platelet maximal aggregation rate–phosphate adenosine; PAg-COL, platelet maximal aggregation rate–collagen; PLT, platelet; PT, prothrombin time; PTA, prothrombin activity; RBC, red blood cell; TC, total cholesterol; TG, triglyceride; TT, prothrombin time; UA, uric acid; UREA, urea; VLDL, very low-density lipoprotein; WBC, white blood cell count; γ-GT, γ-glutamyl transpeptidase.

a *p* < 0.1.

b *p* < 0.5.

c *p* < 0.001.

### Multivariate Statistical Analysis

**Fig. 1:**
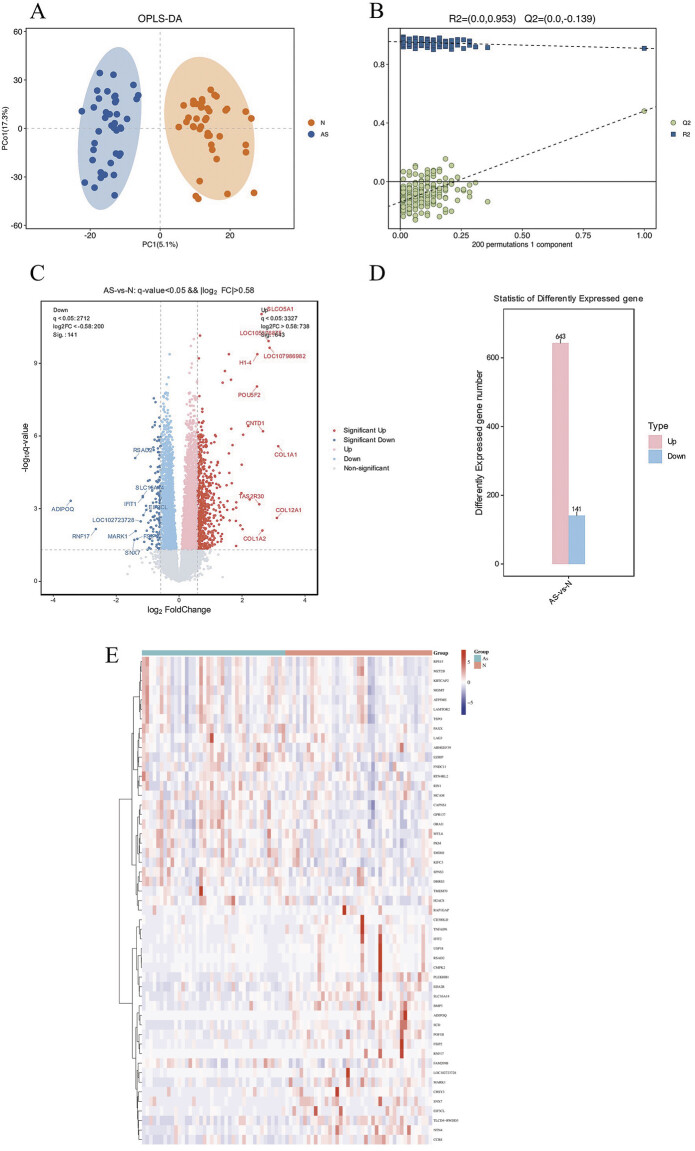
**(A)** OPLS-DA results of the two groups. **(B)** Permutation test of OPLS-DA. **(C)** Volcano map of the DEGs. **(D)** DEGs between the two groups. **(E)** Heatmap of the top 50 DEGs between the groups. DEG, differentially expressed gene; OPLS-DA, orthogonal partial least squares-discriminant analysis.

The transcriptome sequencing results of the two groups were subjected to multivariate statistical analysis, yielding an OPLS-DA score plot, as depicted in Fig. 1A . To assess the potential overfitting of the model, a permutation test was conducted on the OPLS-DA model. The grouping markers for each sample were randomly shuffled prior to modeling and prediction. A robust supervised model requires that the intercept between the regression line (dashed line) at Q2 and the y-axis is less than 0, which indicates satisfactory model quality. As shown in Fig. 1B , the OPLS-DA model employed in this study did not exhibit overfitting and effectively revealed the differences between the two groups of samples.

### Comparative Analysis and Enrichment Assessment of Platelet Transcriptome Findings between the Two Groups

**Fig. 2:**
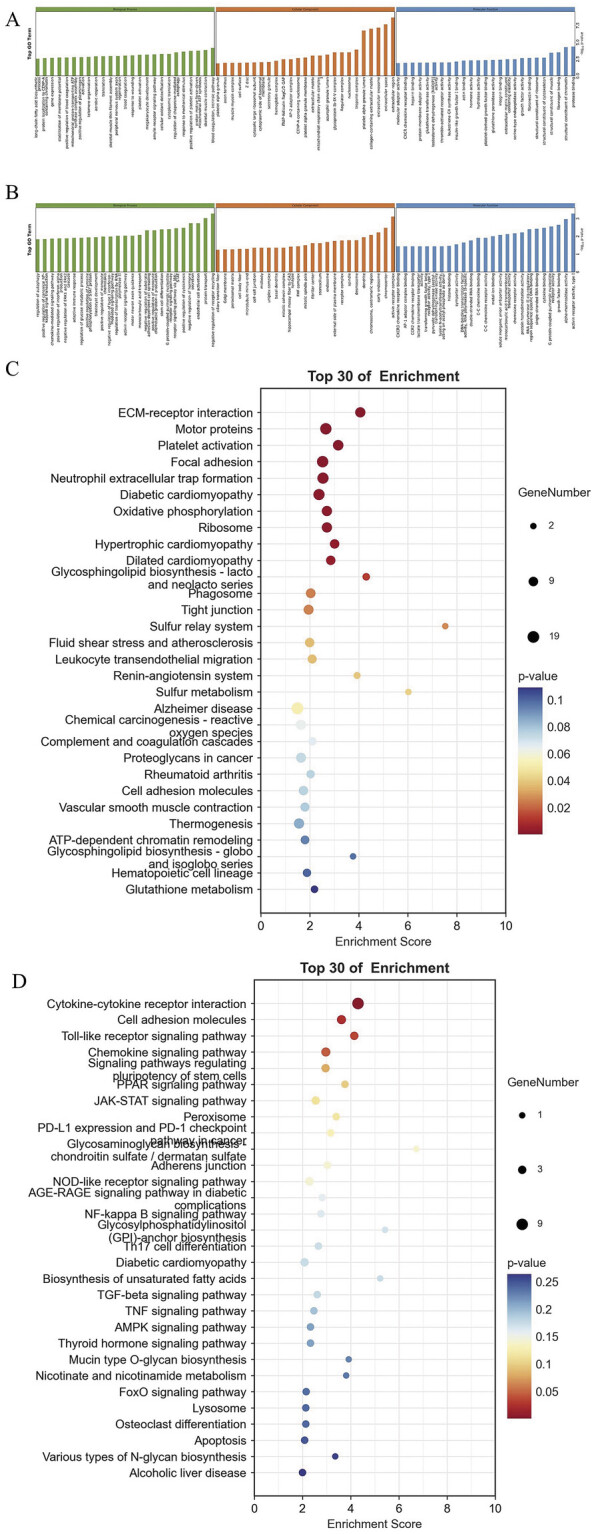
**(A)** GO analysis of upregulated DEGs. **(B)** GO analysis of downregulated DEGs. **(C)** KEGG analysis of upregulated DEGs. **(D)** KEGG analysis of downregulated DEGs. DEG, differentially expressed gene; GO, Gene Ontology; KEGG, Kyoto Encyclopedia of Genes and Genomes.

A total of 784 DEGs, consisting of 141 downregulated genes and 643 upregulated genes, were identified via the DESeq tool ( Fig. 1C , D ). The cluster heatmap visually represented the top 50 DEGs with fold changes between the two groups ( Fig. 1E ). Gene Ontology (GO) enrichment analysis and KEGG signaling pathway enrichment analysis were subsequently performed to further elucidate the specific biological processes associated with these DEGs ( Fig. 2 ).

The GO biological function enrichment analysis indicated that the upregulated DEGs were involved in processes such as blood coagulation, proton motive force-driven mitochondrial ATP synthesis, and positive regulation of platelet activation. Additionally, these genes were significantly enriched in biological processes, including the regulation of chaperone-mediated autophagy (CMA) and megakaryocyte development. Furthermore, the KEGG pathway enrichment analysis revealed that 26 KEGG pathways, including but not limited to extracellular matrix–receptor interaction, platelet activation, focal adhesion, neutrophil extracellular trap formation, fluid shear stress, and AS, were significantly enriched among the upregulated genes.

The downregulated genes were subjected to GO enrichment analysis, which revealed significant associations with negative regulation of receptor recycling, endothelial cell activation, and inhibition of hormone secretion, as well as positive regulation of cytokines and the Janus Kinase–Signal Transducer and Activator (JAK-STAT) signaling pathway. Additionally, KEGG pathway enrichment analysis revealed nine significantly enriched pathways among the downregulated genes, including cytokine–cytokine receptor interactions, cell adhesion molecules, the Toll-like receptor signaling pathway, and the chemokine signaling pathway.

### Hub Genes' Identification through the Integration of Weighted Gene Coexpression Network Analysis

**Fig. 3:**
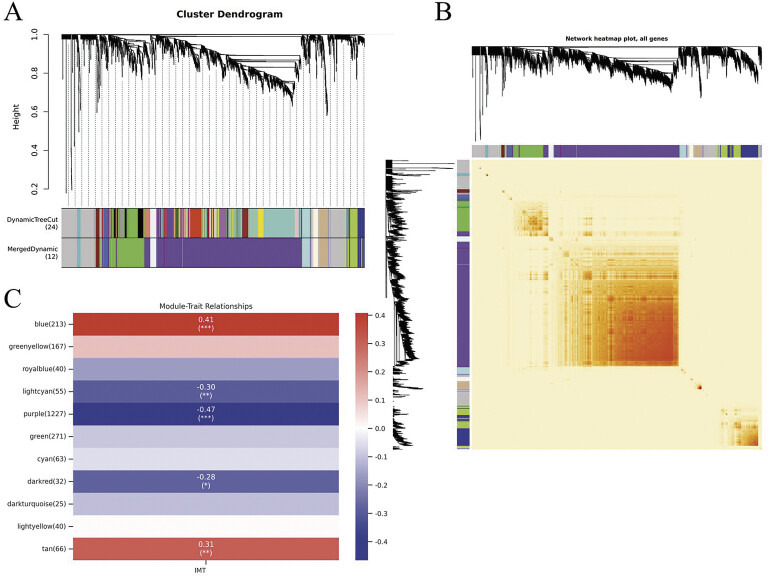
**(A)** Clustering dendrogram of genes, utilizing dissimilarity metrics derived from topological overlay, along with the corresponding assigned module colors. **(B)** Heatmap of intergene correlation based on topological overlapping matrix. **(C)** Correlation and significance between different gene modules and IMT. IMT, intima-media thickness.

**Fig. 4:**
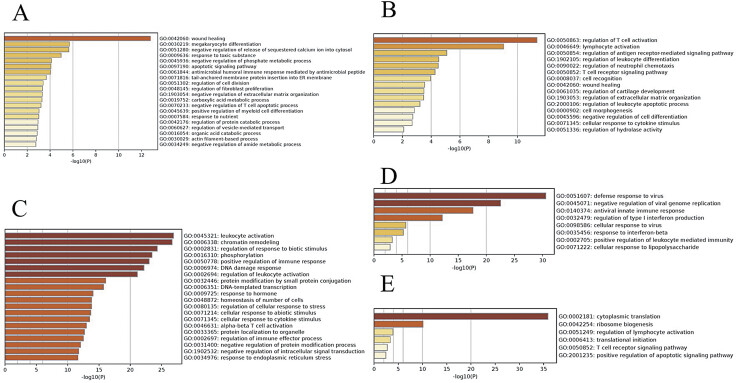
A: GO analysis of the blue module; B: GO analysis of the light cyan module; C: GO analysis of the purple module; D: GO analysis of the dark module; **(E)** GO biological process analysis of the blue, light cyan, purple, dark, and tan modules. GO, Gene Ontology.

We computed the module modules to represent the overall gene expression levels of each module, which were then clustered on the basis of their correlation patterns. A total of 12 modules were identified and assigned unique colors for labeling purposes ( Fig. 3A , B ). A heatmap was subsequently generated to visualize the relationships between modules and traits via Spearman's correlation coefficients, allowing assessment of the association between IMT and the disease under investigation ( Fig. 3C ). Five modules were identified as significantly correlated with IMT. Biological process enrichment analysis of genes across different modules revealed that the blue module is associated with biological processes such as wound healing and megakaryocyte differentiation, whereas the light cyan module pertains to T-cell activation and lymphocyte activation. The purple module is linked to leukocyte activation, whereas the dark module is related to the innate immune response. Finally, the tan module is associated with cytoplasmic translation ( Fig. 4 ).

Among these modules, two (referred to as “purple” and “blue”) exhibited significant associations with AS and were consequently selected as IMT-related modules (purple module: *r* = − 0.47, *p* = 2.22E-05; blue module: *r* = 0.41, *p* = 0.000260805; Supplementary Table S2 ). Notably, while the blue module displayed a positive correlation with IMT encompassing 214 genes, the purple module demonstrated a negative correlation with IMT involving 1,228 genes. These specific genes were retained for subsequent analyses.

**Fig. 5:**
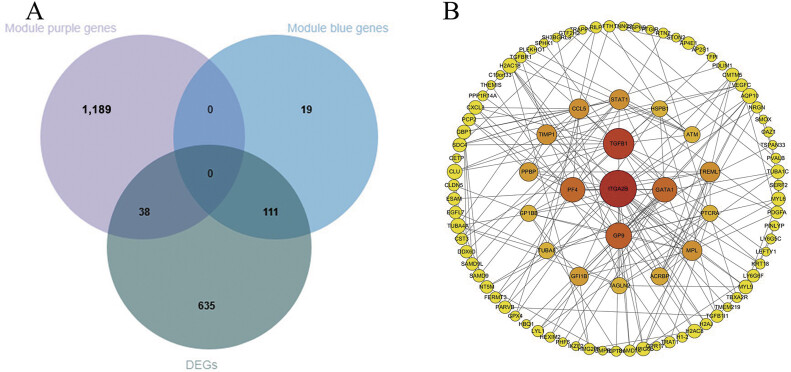
**(A)** Venn diagram of DEGs with purple module and blue module genes. **(B)** PPI network analysis of intersection genes. DEG, differentially expressed gene; PPI, protein–protein interaction.

The DEGs identified earlier were subsequently intersected with the enriched blue and purple module genes. The Venn diagram revealed that there were 111 intersection genes between the DEGs and the blue module, whereas 38 intersection genes were found between the DEGs and the purple module ( Fig. 5A ). Furthermore, a PPI network was constructed from these intersecting genes, leading to the identification of hub genes such as *ITGA2B* , *TGFB1* , *PF4* , *GP9* , and *GATA1* ( Fig. 5B ). Pearson correlation analysis was subsequently conducted to examine the relationships between the hub genes and IMT, thereby validating the findings. The correlation coefficients and *p* -values for the five hub genes related to IMT are presented in Table 4 . *ITGA2B* ( *r* = 0.327), *TGFB1* ( *r* = 0.362), *PF4* ( *r* = 0.240), and *GP9* ( *r* = 0.302) exhibited statistically significant moderate correlations with IMT ( *p* < 0.05).

**Table 4:** Pearson's correlation test between hub genes and intima-media thickness

	Pearson correlation coefficient	*p* -Value
PAg-AA	−0.027	0.817
PAg-ADP	−0.06	0.612
PAg-COL	0.023	0.844
*ITGA2B*	0.327	0.004 a
*TGFB1*	0.362	0.001 a
*PF4*	0.240	0.038 b
*GP9*	0.302	0.008 a
*GATA1*	0.184	0.114

Abbreviations: PAg-AA, platelet maximal aggregation rate–arachidonic acid; PAg-ADP, platelet maximal aggregation rate–phosphate adenosine; PAg-COL, platelet maximal aggregation rate–collagen.

a *p* < 0.1.

b *p* < 0.5.

## Discussion

The prevailing understanding of platelets has been confined to their fundamental role in hemostasis and thrombosis. 5 18 19 20 The utilization of antiplatelet medications such as thromboxane A2 inhibitors and adenosine diphosphate purinergic receptor P2Y, G-Protein coupled, 12 (P2Y12) inhibitors for the prevention and treatment of AS and thrombosis has resulted in promising outcomes. 21 22 23 Over the past decade, our understanding of platelet pathophysiology has undergone significant updates 24 25 ; however, the precise involvement of platelets in AS pathogenesis remains unknown. Intriguingly, this study did not reveal any notable statistical disparity in platelet aggregation parameters between the two groups, implying that current platelet aggregation detection methods may not be effective for the discovery of AS. Consequently, we used transcriptome sequencing technology to provide a comprehensive understanding of the role of platelets in AS development, thereby offering novel insights into the development of more sensitive platelet biomarkers and safer antiplatelet therapies.

The analysis of DEGs revealed 141 downregulated genes and 643 upregulated genes in patients with AS. GO and KEGG analyses revealed that these DEGs were not only enriched in traditional platelet-related biological processes such as blood coagulation and platelet activation but also significantly enriched in pathways related to Toll-like receptor signaling, chemokine signaling, cytokine–cytokine receptor interaction, and neutrophil extracellular trap formation. Similarly, among the five gene modules identified by Weighted Gene Coexpression Network Analysis (WGCNA) that were significantly associated with IMT, the enrichment of genes in three modules indicates T-cell activation (light cyan module), lymphocyte activation (light cyan module), and biological processes such as leukocyte activation (purple module) and the innate immune response (dark module). These findings suggest a close association between the role of platelets in AS pathogenesis and inflammation status. As previous research has shown, AS is fundamentally characterized by chronic inflammation involving various effector cells, including neutrophils, monocytes, and lymphocytes. 26 27 On one hand, activated platelets secrete various cytokines and chemokines to facilitate immune cell recruitment to the lesion site. 28 29 30 31 On the other hand, there is a complex interplay between platelets and diverse inflammatory effector cells that collectively regulate the inflammatory response, thereby exerting control over the onset and progression of AS. 9 10 11 12 28 32

The correlations between the identified central genes and the severity of IMT were analyzed, revealing positive associations of *ITGA2B* ( *r* = 0.327, *p* = 0.004), *TGFB1* ( *r* = 0.362, *p* = 0.001), *PF4* ( *r* = 0.240, *p* = 0.038), and *GP9* ( *r* = 0.302, *p* = 0.008) with IMT. *Platelet Factor 4 (PF4)* , also referred to as CXCL4 (C-X-C motif chemokine ligand 4), encodes a member of the CXC chemokine family. 33 In the present study, *PF4* was upregulated in patients with AS, which is consistent with prior publications. This chemokine is released from activated platelet alpha granules in the form of a homotetramer with a strong affinity for heparin and plays a role in platelet aggregation. 34 Moreover, this protein has chemotactic effects on various cell types and acts as an inhibitor of hematopoiesis, angiogenesis, and T-cell function. 35 Previous studies have demonstrated the proatherogenic effects of *PF4* through its ability to form heterodimers and oligomers with CCL5, leading to CXCL4-induced monocyte binding to endothelial cells and subsequent monocyte exudation into the subendothelial space. 36 37 38 Additionally, CXCL4 influences monocyte differentiation by inducing a specific macrophage phenotype known as M4, a proinflammatory marker associated with plaque instability. 36 Furthermore, *PF4* serves as an important mediator in regulating T-cell differentiation related to platelet function and participates in thrombosis progression. 39 Combined with our study, *PF4* may be a potential platelet biomarker for AS.

*GP9* represents another differentially expressed hub gene encoding a small membrane glycoprotein located on the surface of human platelets. 40 It forms a non-covalent complex with glycoprotein Ib, which is part of a platelet surface membrane glycoprotein complex that serves as a receptor for von Willebrand factor. 41 The complete receptor complex comprises the non-covalent association of alpha and beta subunits with the protein encoded by this gene, along with platelet glycoprotein V. 42 Currently, research concerning *GP9* primarily focuses on Bernard–Soulier syndrome, 43 44 45 and no association with AS status has been documented. A study conducted by Burkard et al. demonstrated that glycoprotein VI exacerbates lipopolysaccharide-induced acute lung injury in mice through stimulation of neutrophil extracellular trap formation. 46 In the present study, KEGG pathway analysis revealed significant enrichment of upregulated genes within the neutrophil extracellular trap formation pathway, potentially providing insiPghts for further investigation into the mechanisms underlying platelet involvement in AS development.

Similarly, TGFB1 also showed a high correlation with IMT. The involvement of TGFβ in various biological processes, including cell proliferation, differentiation, migration, adhesion, and extracellular matrix production, has been extensively acknowledged. 47 Notably, the TGFβ signaling signature and the expression profiles of TGFβ ligands, receptors, and diverse Smad proteins have been documented in atherosclerotic plaques. 48 49 Chen et al. identified endothelial TGFβ signaling as one of the primary drivers of vascular inflammation associated with AS. 50 Platelets contain substantial amounts of TGFβ1 51 ; however, there are currently no published studies detailing the specific mechanisms by which platelet-derived TGFβ1 regulates the pathogenesis of AS.

In the present study, we identified associations between carotid IMT and the hub genes *ITGA2B* , *TGFB1* , *PF4* , and *GP9* through WGCNA and correlation analysis, and these findings are novel. These hub genes may serve as platelet biomarkers that could aid in the prediction and diagnosis of AS; however, further validation in a larger cohort is necessary.

Furthermore, our study presents several compelling new findings. The upregulated DEGs were significantly enriched in the regulation of CMA. While autophagy has been demonstrated to occur in platelets, this finding contrasts with conclusions drawn from previous studies. 52 53 Qiao et al. reported that CMA function is compromised during the progression of AS, leading to increased activation of the NOD-like receptor family pyrin domain containing 3 (NLRP3) inflammasome and secretion of IL-1β, thereby promoting vascular inflammation and advancing AS. 54 55 However, these observations may be influenced by varying stages of AS or differences in model species, necessitating further validation through more rigorous experimental designs.

In addition to the aforementioned platelet-related information, our laboratory examination of the two cohorts revealed significant between-group differences in RBC and HGB levels, which constitutes a noteworthy finding. Prior studies have shown that the structure and function of RBCs are influenced by circulating plasma LDL-C levels 56 ; furthermore, abnormal collisions of RBCs with arterial walls can lead to endothelial damage, lipid retention, and localized hemolysis, resulting in the release of toxic heme iron—an aspect contributing to the complex pathogenesis of AS. 57 While our findings aligned with those of previous reports, we observed only variations in RBC count and HGB levels between the two groups without delving into their underlying implications.

In recent years, increasing attention has been focused on the role of platelets in the development of AS. While traditional AS biomarkers, such as lipid profiles and imaging techniques, remain widely used, emerging evidence suggests that platelet activation and the identification of platelet-related biomarkers may offer more accurate diagnostic and prognostic capabilities. In addition to the present study, several other studies have explored the role of platelet biomarkers and platelet receptors in the progression and prognosis of AS. For example, a study identified specific platelet receptors associated with the risk of atherosclerotic plaque rupture. 58 Similarly, another demonstrated the inflammatory role of platelet-derived chemokines in acute coronary syndrome and their prognostic value. 59 These findings further support the potential of platelet biomarkers in enhancing the early diagnostic accuracy and therapeutic efficacy of AS.

## Conclusion

In conclusion, our study provides new insights into the role of platelet biomarkers in the diagnosis and progression of AS. Through RNA sequencing of platelets from AS patients, we identified key genes, including *ITGA2B* , *TGFB1* , *PF4* , and *GP9* , whose expression levels were significantly correlated with IMT, suggesting their potential as novel diagnostic biomarkers for AS. While current diagnostic methods, such as lipid profiles and carotid artery ultrasounds, are widely used, our findings highlight the limitations of these traditional techniques and the need for more sensitive biomarkers. The results of this study not only contribute to the understanding of platelet-related mechanisms in AS but also open potential avenues for future clinical applications in early diagnosis and personalized treatment strategies. Further research is needed to validate these biomarkers in larger patient cohorts and explore their clinical utility in real-world settings.

## Notes

**What Is Known About This Topic?**
The utilization of antiplatelet medications such as thromboxane A2 inhibitors and adenosine diphosphate P2Y12 inhibitors for the prevention and treatment of AS and thrombosis has resulted in promising outcomes; however, the specific mechanism of platelet involvement in the pathogenesis of AS is still ambiguous.
**What Does This Paper Add?**

We conducted an RNA-seq analysis to examine the differences in platelet gene expression between healthy individuals and patients with AS. By utilizing bioinformatics tools, we successfully identified four potential platelet biomarkers that could potentially aid in the diagnosis and monitoring of AS progression. These biomarkers include *ITGA2B* ( *r* = 0.327, *p* = 0.004), *TGFB1* ( *r* = 0.362, *p* = 0.001), *PF4* ( *r* = 0.240, *p* = 0.038), and *GP9* ( *r* = 0.302, *p* = 0.008).
