# Supplementary material for: Platelet RNA-Seq Reveals Genes Associated with Carotid Intima-Media Thickness: A Cross-Sectional Study
Source: TH Open. 2025 Aug 7;9:a26616472. doi: 10.1055/a-2661-6472 (PMC12371662; doi:10.1055/a-2661-6472)
Supplement: Supplementary file 1 — Supplementary Material [file 10-1055-a-2661-6472_26789311.pdf]

Supplementary Table 1 The carotid ultrasound results of the participants included in the study

	IMT-LEFT （mm）	IMT-RIGHT （mm）	Plaque size - Left （mm）	Plaque size - Right （mm）
**N1**	0.69	0.49		
**N2**	0.55	0.55		
**N3**	0.42	0.49		
**N4**	0.56	0.56		
**N5**	0.56	0.61		
**N6**	0.47	0.49		
**N7**	0.42	0.49		
**N8**	0.56	0.56		
**N9**	0.56	0.49		
**N10**	0.7	0.63		
**N11**	0.49	0.42		
**N12**	0.49	0.63		
**N13**	0.47	0.47		
**N14**	0.49	0.47		
**N15**	0.47	0.42		
**N16**	0.77	0.9		
**N17**	0.1	0.79		
**N18**	0.69	0.67		
**N19**	0.55	0.55		

TH Open | DOI 10.1055/a-2661-6472 | © The Author(s).

**N20**	0.77	0.76
**N21**	0.49	0.56
**N22**	0.55	0.49
**N23**	0.4	0.63
**N24**	0.55	0.56
**N25**	0.56	0.7
**N26**	0.67	0.67
**N27**	0.55	0.55
**N28**	0.63	0.63
**N29**	0.61	0.55
**N30**	0.55	0.55
**N31**	0.63	0.47
**N32**	0.47	0.42
**N33**	0.61	0.61
**N34**	0.69	0.63
**N35**	0.69	0.61
**N36**	0.56	0.61
**N37**	0.56	0.61
**N38**	0.61	0.56
**N39**	0.77	0.67
**N40**	0.56	0.55
**N41**	0.56	0.56

**As1**	1.12	1.04	11.96*2.46	9.34*3.3
**As2**	0.64	0.07	9.23*3.06	
**As3**	1.04	1.28		9.12*1.71
**As4**	1.46	1.42	9.16*2.06	10.63*2.15
**As5**			10.2*3.1	7.8*1.8
**As6**		1.3		
**As7**		1.1		
**As8**	0.93	1.39		
**As9**	1.33	1.13	12.99*2.4	10.44*3.19
**As10**			10.9*2.9	2.1*1.1
**As11**	1.1	1.1		
**As12**	1.1			
**As13**	1.1	1.1		
**As14**	1.04	1.03		
**As15**				2.7*1.7
**As16**	1.2	1.25	14.83*2.33	
**As17**	1.34	0.56	10.83*2.13	13.98*2.79
**As18**	1.1	1		
**As19**	0.65	0.98		8.51*2.38
**As20**	0.98	1.04		10.07*2.16
**As21**		1.01	10.13*1.95	
**As22**	1.18	1.04	9.76*1.88	9.45*2.5

**As23**		1.3		
**As24**		1.2		
**As25**	1.3	1.2		
**As26**	1.1			
**As27**			4.9*1.8	
**As28**			12.6*1.6	9.5*1.8
**As29**	1.1			
**As30**		1		
**As31**	1.2			
**As32**	1.2			
**As33**		1.2		
**As34**	0.63	0.67	10.06*2.5	10.14*1.82
**As35**	0.61	0.63	5.46*1.52	
**As36**	1.11	1.18	10.55*1.83	
**As37**	1.12	1.11		
**As38**	0.49	1.08		
**As39**	0.49	1.32		
**As40**	0.56	0.56	10.61*1.95	9.55*2.43

Supplementary Table 2 The r-values and p-values of the different color modules identified by WGCNA

Module	r-values	p-values
blue	0.407281	0.000261***
greenyellow	0.109373	0.346953
royalblue	-0.16417	0.156452
lightcyan	-0.29673	0.009246**
purple	-0.46593	0.0000222***
green	-0.09246	0.426965
cyan	-0.05326	0.647707
darkred	-0.27851	0.014842*
darkturquoise	-0.10558	0.364030
lightyellow	0.006375	0.956415
tan	0.310726	0.006296**

***$p < 0.001$

**$p < 0.1$

*$p < 0.5$
